# Supplementary material for: Breastfeeding and Prevalence of Metabolic Syndrome among Perimenopausal Women
Source: Nutrients. 2020 Sep 3;12(9):2691. doi: 10.3390/nu12092691 (PMC7551428; doi:10.3390/nu12092691)
Supplement: Supplementary file 1 [file nutrients-12-02691-s001.pdf]

Table S1. Baseline characteristics of nulliparous and parous women (n = 7621).

**Variables**		Nulliparous n = 597	Parous n = 7024	p
**Age (years), X (SD)**		**55.88 (3.15)**	**55.36 (5.32)**	**0.023**
Years of education, X (SD)		**14.55 (3.04)**	**13.15 (3.15)**	**<0.001**
Place of living, N (%)	City	**465 (77.89)**	**4319 (61.49)**	**<0.001**
	Village	**132 (22.11)**	**2705 (38.51)**	
Marital status, N (%)	Single	**398 (66.67)**	**1529 (21.77)**	**<0.001**
	Married	**199 (33.33)**	**5495 (78.23)**	
Menopausal status, N (%)	Premenopausal	154 (25.80)	1987 (28.29)	0.193
	Postmenopausal	443 (74.20)	5037 (71.71)	
Hormone therapy, N (%)	No	**547 (91.62)**	**6119 (87.12)**	**0.001**
	Yes	**50 (8.38)**	**905 (12.88)**	
Smoking, N (%)	No	309 (51.76)	3703 (52.72)	0.652
	Yes	288 (48.24)	3321 (47.28)	
Traditional-carbohydrate DP, N (%)	T1 – the lowest	183 (30.65)	2355 (33.53)	0.245
	T2	215 (36.01)	2322 (33.06)	
	T3 – the highest	199 (33.33)	2347 (33.41)	
Healthy DP, N (%)	T1 – the lowest	**249 (41.71)**	**2289 (32.59)**	**<0.001**
	T2	**171 (28.64)**	**2367 (33.70)**	
	T3 – the highest	**177 (29.65)**	**2368 (33.71)**	
Westernized, DP N (%)	T1 – the lowest	**239 (40.03)**	**2299 (32.73)**	**<0.001**
	T2	**202 (33.84)**	**2299 (32.73)**	
	T3 – the highest	**156 (26.13)**	**2390 (34.03)**	
Physical activity (MET/day/min^{-1}), X (SD)		**516.51 (426.44)**	**608.71 (481.58)**	**<0.001**
Sitting time (min/day), X (SD)		**321.68 (142.67)**	**292.55 (133.63)**	**<0.001**
BMI [kg/m^2], X (SD)		**27.08 (5.02)**	**28.03 (4.93)**	**<0.001**
Metabolic syndrome, N (%)	No	347 (58.12)	4058 (57.77)	0.868
	Yes	250 (41.88)	2966 (42.23)	
Abdominal obesity, N (%)	No	**187 (31.32)**	**1699 (24.19)**	**<0.001**
	Yes	**410 (68.68)**	**5325 (75.81)**	
Increased glucose concentration, N (%)	No	430 (72.03)	5117 (72.85)	0.664
	Yes	167 (27.97)	1907 (27.15)	
Elevated blood pressure, N (%)	No	173 (28.98)	2130 (30.32)	0.492
	Yes	424 (71.02)	4894 (69.68)	
Increased triglyceride concentration, N (%)	No	404 (67.67)	4872 (69.36)	0.390
	Yes	193 (32.33)	2152 (30.64)	
Decreased HDL-cholesterol concentration, N (%)	No	406 (68.01)	4881 (69.49)	0.450

Yes	191 (31.99)	2143 (30.51)

T – tertile; — numbers in bold indicate statistically significant results.

Table S2. Baseline characteristics of parous women in relation to breastfeeding status (*n* = 7024).

**Variables**		Never breastfed *n* = 767 (0)	Breastfed 1–6 months *n* = 2284 (1)	Breastfed 7–12 months *n* = 1626 (2)	Breastfed 13–18 months *n* = 817 (3)	Breastfed >18 months *n* = 1530 (4)	*p*
**Age (years), X (SD)**		**55.95 (4.93)**	**55.55 (5.12)**	**55.54 (5.40)**	**55.37 (5.36)**	**54.61 (5.64)**	**<0.001**
Years of education, X (SD)		**13.45 (3.01)**	**13.53 (2.97)**	**13.00 (3.09)**	**13.13 (3.24)**	**12.59 (3.40)**	**<0.001**
Place of living, N (%)	City	**542 (70.66)**	**1587 (69.48)**	**990 (60.89)**	**464 (56.79)**	**736 (48.10)**	**<0.001**
	Village	**225 (29.34)**	**697 (30.52)**	**636 (39.11)**	**353 (43.21)**	**794 (51.90)**	
Marital status, N (%)	Single	**205 (26.73)**	**550 (24.08)**	**326 (20.05)**	**169 (20.69)**	**279 (18.24)**	**<0.001**
	Married	**562 (73.27)**	**1734 (75.92)**	**1300 (79.95)**	**648 (79.31)**	**1251 (81.76)**	
Menopausal status, N (%)	Premenopausal	**189 (24.64)**	**581 (25.440**	**452 (27.80)**	**226 (27.66)**	**539 (35.23)**	**<0.001**
	Postmenopausal	**578 (75.36)**	**1703 (74.56)**	**1174 (72.20)**	**591 (72.34)**	**991 (64.77)**	
Parity, N (%)	1	**277 (36.11)**	**649 (28.42)**	**162 (9.96)**	**40 (4.90)**	**49 (3.20)**	**<0.001**
	2	**372 (48.50)**	**1321 (57.84)**	**973 (59.84)**	**375 (45.20)**	**493 (32.22)**	
	3 and more	**118 (15.38)**	**314 (13.75)**	**491 (30.20)**	**402 (49.20)**	**988 (64.58)**	
Hormone therapy, N (%)	No	**109 (14.21)**	**333 (14.58)**	**202 (12.42)**	**107 (13.10)**	**154 (10.07)**	**0.001**
	Yes	**658 (85.79)**	**1951 (85.42)**	**1424 (87.58)**	**710 (86.90)**	**1376 (89.93)**	
Smoking, N (%)	No	**361 (47.07)**	**1076 (47.11)**	**869 (53.44)**	**464 (56.79)**	**933 (60.98)**	**<0.001**
	Yes	**406 (52.93)**	**1208 (52.89)**	**757 (46.56)**	**353 (43.21)**	**597 (39.02)**	
Traditional-carbohydrate DP, N (%)	T1 – the lowest	238 (31.03)	814 (35.64)	538 (33.09)	266 (32.56)	499 (32.61)	0.076
	T2	287 (37.42)	735 (32.18)	528 (32.47)	277 (33.90)	495 (32.35)	
	T3 – the highest	242 (31.55)	735 (32.18)	560 (34.44)	274 (33.54)	536 (35.03)	
Healthy DP, N (%)	T1 – the lowest	257 (33.51)	735 (32.18)	533 (32.78)	268 (32.80)	496 (32.42)	0.554
	T2	279 (36.38)	750 (32.84)	542 (33.33)	280 (34.27)	516 (33.73)	
	T3 – the highest	231 (30.12)	799 (34.98)	551 (33.89)	269 (32.93)	518 (33.86)	
Westernized DP, N (%)	T1 – the lowest	**289 (37.68)**	**842 (36.87)**	**502 (30.87)**	**244 (29.87)**	**422 (27.58)**	**<0.001**
	T2	**254 (33.12)**	**741 (32.44)**	**548 (33.70)**	**287 (35.13)**	**505 (33.01)**	
	T3 – the highest	**224 (29.20)**	**701 (30.69)**	**576 (35.42)**	**286 (35.01)**	**603 (39.41)**	
Physical activity (MET/day/min^{-1}), X (SD)		**523.37 (416.31)**	**557.74 (443.25)**	**621.65 (489.71)**	**618.53 (459.69)**	**708.58 (547.43)**	**<0.001**
Sitting time (min/day), X (SD)		**297.13 (135.21)**	**298.66 (134.05)**	**291.65 (132.97)**	**293.70 (135.32)**	**282.16 (131.46)**	**0.008**
BMI [kg/m^2], X (SD)		**28.08 (5.15)**	**27.43 (4.63)**	**28.02 (4.76)**	**28.27 (5.11)**	**28.78 (5.20)**	**<0.001**
Metabolic syndrome, N (%)	No	**408 (53.19)**	**1349 (59.06)**	**927 (57.01)**	**486 (59.49)**	**888 (58.04)**	**0.049**

	Yes	**359 (46.81)**	**935 (40.94)**	**699 (42.99)**	**331 (40.51)**	**642 (41.96)**	
Abdominal obesity, N (%)	No	**181 (23.60)**	**649 (28.42)**	**360 (22.14)**	**185 (22.64)**	**324 (21.18)**	**<0.001**
	Yes	**586 (76.40)**	**1635 (71.58)**	**1266 (77.86)**	**632 (77.36)**	**1206 (78.82)**	
Increased glucose concentration, N (%)	No	531 (69.23)	1680 (73.56)	1211 (74.48)	587 (71.85)	1108 (72.42)	0.078
	Yes	236 (30.77)	604 (26.44)	415 (25.52)	230 (28.15)	422 (27.58)	
Elevated blood pressure, N (%)	No	224 (29.20)	663 (29.03)	511 (31.43)	255 (31.21)	477 (31.18)	0.401
	Yes	543 (70.80)	1621 (70.97)	1115 (68.57)	562 (68.79)	1053 (68.82)	
Increased triglyceride concentration, N (%)	No	**505 (65.84)**	**1604 (70.23)**	**1102 (67.77)**	**583 (71.36)**	**1078 (70.46)**	**0.048**
	Yes	**262 (34.16)**	**680 (29.77)**	**524 (32.23)**	**234 (28.64)**	**452 (29.54)**	
Decreased HDL-cholesterol concentration, N (%)	No	518 (67.54)	1600 (70.05)	1122 (69.00)	580 (70.99)	1061 (69.35)	0.594
	Yes	249 (32.46)	684 (29.95)	504 (31.00)	237 (29.01)	469 (30.65)	

T – tertile; — numbers in bold indicate statistically significant results.

Table S3. Comparisons between categories of total breastfeeding duration - the results of post-hoc tests.

	0 vs. 1	0 vs. 2	0 vs. 3	0 vs. 4	1 vs. 2	1 vs. 3	1 vs. 4	2 vs. 3	2 vs. 4	3 vs. 4
Age	$p < 0.05$	$p < 0.05$	$p < 0.05$	$p < 0.05$	-	-	-	-	-	-
Education	-	$p < 0.05$	-	$p < 0.05$	$p < 0.05$	$p < 0.05$	$p < 0.05$	-	$p < 0.05$	$p < 0.05$
Physical activity	-	$p < 0.05$	$p < 0.05$	$p < 0.05$	$p < 0.05$	$p < 0.05$	$p < 0.05$	-	-	$p < 0.05$
Sitting time	-	-	-	$p < 0.05$	-	-	-	-	-	-
BMI	-	-	-	$p < 0.05$	$p < 0.05$	$p < 0.05$	$p < 0.05$	-	$p < 0.05$	-

Table S4. Multivariable logistic regression analysis for MetS and its components in relation to breastfeeding duration (unadjusted).

Breastfeeding status	Metabolic Syndrome		Abdominal obesity		Increased glucose concentration		Elevated blood pressure		Increased triglyceride concentration		Decreased HDL-cholesterol concentration	
	OR (95% CI)	*p*	OR (95% CI)	*p*	OR (95% CI)	*p*	OR (95% CI)	*p*	OR (95% CI)	*p*	OR (95% CI)	*p*
Never	1.00		1.00		1.00		1.00		1.00		1.00	
Ever breastfed	**0.81 (0.70–0.94)**	**0.006**	0.96 (0.81–1.15)	0.686	**0.82 (0.70–0.97)**	**0.017**	0.94 (0.80–1.11)	0.475	**0.83 (0.71–0.98)**	0.025	0.90 (0.77–1.06)	0.213
Breastfed 1–6 months	**0.79 (0.67–0.93)**	**0.005**	**0.78 (0.64–0.94)**	**0.010**	**0.81 (0.68–0.97)**	**0.021**	1.01 (0.84–1.21)	0.926	**0.82 (0.69–0.97)**	**0.023**	0.89 (0.75–1.06)	0.493
7–12 months	0.86 (0.72–1.02)	0.080	1.09 (0.89–1.33)	0.426	**0.77 (0.63–0.93)**	**0.007**	0.90 (0.75–1.09)	0.272	0.92 (0.76–1.10)	0.348	0.93 (0.78–1.12)	0.713
13–18 months	**0.77 (0.63–0.94)**	**0.012**	1.06 (0.83–1.33)	0.652	0.88 (0.71–1.09)	0.253	0.91 (0.73–1.13)	0.385	**0.77 (0.62–0.96)**	**0.018**	0.85 (0.69–1.05)	0.249
>18 months	**0.82 (0.69–0.98)**	**0.027**	1.15 (0.93–1.41)	0.186	0.86 (0.71–1.04)	0.111	0.91 (0.75–1.10)	0.333	**0.81 (0.67–0.97)**	**0.024**	0.92 (0.76–1.11)	0.962

Numbers in bold indicate statistically significant results.

Table S5. Multivariable logistic regression analysis for MetS and its components in relation to parity and breastfeeding duration (unadjusted).

Parity	Breastfeeding status	Metabolic Syndrome		Abdominal obesity		Increased glucose concentration		Elevated blood pressure		Increased triglyceride concentration		Decreased HDL-cholesterol concentration	
		OR (95% CI)	*p*	OR (95% CI)	*p*	OR (95% CI)	*p*	OR (95% CI)	*p*	OR (95% CI)	*p*	OR (95% CI)	*p*
**Nulliparous**	-	1.00		1.00		1.00		1.00		1.00		1.00	
Parous	-	1.01 (0.86–1.2)	0.868	**1.43 (1.19–1.71)**	**<0.001**	0.93 (0.78–1.12)	0.450	0.94 (0.78–1.13)	0.492	0.92 (0.77–1.11)	0.390	0.93 (0.78–1.12)	0.450
One child	Never	1.00		1.00		1.00		1.00		1.00		1.00	
	1–6 months	0.98 (0.74–1.31)	0.893	0.94 (0.70–1.27)	0.699	0.80 (0.58–1.10)	0.161	1.26 (0.93–1.70)	0.130	0.95 (0.70–1.29)	0.726	1.10 (0.81–1.50)	0.545
	7–12 months	0.98 (0.66–145)	0.907	1.12 (0.74–1.71)	0.585	0.73 (0.46–1.15)	0.176	0.89 (0.59–1.33)	0.562	1.05 (0.69–1.59)	0.819	1.06 (0.69–1.62)	0.805
	13–18 months	0.81 (0.40–1.61)	0.540	0.71 (0.36–1.40)	0.324	1.42 (0.71–2.87)	0.323	1.08 (0.53–2.20)	0.823	**0.39 (0.16–0.97)**	**0.042**	0.53 (0.23–1.25)	0.148
	>18 months	**0.48 (0.24–0.97)**	**0.041**	0.75 (0.40–1.40)	0.363	0.59 (0.28–1.28)	0.186	0.82 (0.44–1.54)	0.545	0.64 (0.31–1.32)	0.228	0.73 (0.35–1.49)	0.382
Two children	Never	1.00		1.00		1.00		1.00		1.00		1.00	
	1–6 months	1.01 (0.75–1.37)	0.935	0.97 (0.71–1.32)	0.839	0.79 (0.57–1.11)	0.176	1.32 (0.97–1.80)	0.078	1.00 (0.73–1.38)	0.976	1.13 (0.82–1.56)	0.456
	7–12 months	1.00 (0.66–1.52)	0.996	1.17 (0.75–1.81)	0.483	0.73 (0.45–1.17)	0.191	0.90 (0.59–1.37)	0.631	1.07 (0.70–1.66)	0.748	1.03 (0.66–1.61)	0.884
	13–18 months	1.07 (0.51–2.27)	0.850	0.93 (0.45–1.89)	0.835	1.85 (0.88–3.88)	0.104	1.43 (0.68–3.01)	0.345	0.45 (0.18–1.16)	0.098	0.62 (0.25–1.51)	0.288
	>18 months	0.65 (0.31–1.35)	0.246	0.94 (0.49–1.82)	0.863	0.80 (0.36–1.79)	0.593	0.98 (0.51–1.14)	0.944	0.88 (0.42–1.87)	0.744	0.92 (0.43–1.96)	0.834
Three or more children	Never	1.00		1.00		1.00		1.00		1.00		1.00	
	1–6 months	**0.59 (0.38–0.90)**	**0.015**	**0.40(0.21–0.76)**	**0.005**	0.77 (0.50–1.21)	0.258	0.84 (0.51–1.36)	0.473	**0.63 (0.40–0.97)**	**0.038**	0.87 (0.55–1.36)	0.532
	7–12 months	0.76 (0.51–1.14)	0.186	0.60 (00.32–1.12)	0.108	0.66 (0.44–1.01)	0.057	0.71 (0.45–1.12)	0.139	0.81 (0.54–1.23)	0.330	1.00 (0.65–1.53)	0.991
	13–18 months	**0.62 (0.41–0.93)**	**0.022**	0.62 (0.33–1.17)	0.138	0.69 (0.45–1.06)	0.092	0.72 (0.45–1.15)	0.174	**0.59 (0.39–0.91)**	**0.016**	0.80 (0.52–1.25)	0.326
	>18 months	**0.66 (0.45–0.96)**	**0.031**	0.56 (0.31–1.02)	0.057	**0.67 (0.45–0.99)**	**0.050**	0.73 (0.47–1.13)	0.161	**0.65 (0.44–0.97)**	**0.033**	0.94 (0.63–1.41)	0.763

Numbers in bold indicate statistically significant results.
